# Fluorescent antimicrobial hydrogel based on fluorophore N-doped carbon dots originated from cellulose nanocrystals

**DOI:** 10.1038/s41598-024-80222-7

**Published:** 2024-11-25

**Authors:** Hanan B. Ahmed, Hossam E. Emam, Tharwat I. Shaheen

**Affiliations:** 1 Chemistry Department, Faculty of Science, Helwan University, Ain-Helwan, Cairo, 11795 Egypt; 2 Department of Pretreatment and Finishing of Cellulosic Based Textiles, Textile Research and Technology Institute, National Research Centre, Scopus Affiliation ID 60014618, 33 EL Buhouth St., Dokki, Giza, 12622 Egypt

**Keywords:** Cellulose nanocrystals, Carbon dots, N-carbon dots, Hydrogel, Fluorescent, Antimicrobial, Quantum dots, Biopolymers

## Abstract

The current study represents a unique fabrication strategy for preparation of fluorescent hydrogels via incorporation of fluorescent quantum dots (QDs) as fluorophore entities into chitosan as a gelling matrix. QDs identified as carbon quantum dots (CQDs) & nitrogen containing carbon quantum dots (NCQDs) were preliminary synthesized from cellulose nanocrystals (CNCs) and cationic cellulose nanocrystals (CCNCs), respectively. Cationic CNCs was prepared via chemical grafting with poly-di-allyl dimethyl ammonium chloride (CNCs-g-poly-DADMAC) through free chain polymerization reaction. Additionally, both of the prepared CQDs & NCQDs were impregnated in 3D interpenetrating network of chitosan for preparation of microbicide/florescent hydrogels (CQDs@Chs hydrogel & NCQDs@Chs hydrogel). The represented data revealed that, exploitation of cationic CNCs resulted in preparation of NCQDs with more controllable size and superior photoluminescence. Moreover, the increment in concentration of CNCs reflected in nucleation of enlarged QDs, at variance of CCNCs, whereas, increment of concentration resulted in significantly smaller-sized QDs. Size distribution of CQDs ingrained from 2% CNCs was estimated to be 8.2 nm, while, NCQDs ingrained from 2% CCNCs exhibited with size distribution of 3.8 nm. The prepared florescent CQDs@Chs hydrogel & NCQDs@Chs hydrogel showed excellent antimicrobial performance and the diameter of inhibition zone was estimated to be 31 mm, 26 mm & 22 mm against *E. Coli, S. Aureus & C. Albicans* with CQDs@Chs, respectively. Whereas, treatment of the as-mentioned microbial strains with NCQDs@Chs resulted in detection of inhibition zone diameter to be significantly higher as 34 mm, 28 mm & 25 mm for *E. Coli, S. Aureus & C. Albicans*, respectively. In a conclusion, cationic CNCs showed seniority in nucleation of QDs with significantly higher photoluminescence and microbicide activities.

**Supplementary Information:**

The online version contains supplementary material available at 10.1038/s41598-024-80222-7.

## Introduction

Several types of biopolymers were shown to exhibit superior applicability in various biomedical purposes^1,2^. Cellulose nano-whiskers (CNCs), also identified as cellulose whiskers, cellulose nanocrystals, and nanocrystalline cellulose are characterized with nano-scaled width and length dimensions^3^. Bacterial cellulose, tunicate, ramie, cotton, soft wood and hard wood, could be exploited as cellulosic resources. CNCs can be clustered from the naturally occurred cellulose via acidic or enzymatic hydrolysis^4^. Despite of the sources of cellulose nano-whiskers are different, the rod-like CNCs exhibit a high surface area with decorative hydroxyl groups that give CNCs some superior characters, such as high crystallinity, high reactivity, high strength and young’s modulus, hyperfine composition, high transparency, and high purity^5–7^. These characters are significantly differing from those of the regular cellulosic fibers, which enables CNCs to be exploited for widespread applications^8–10^.

Attributing to the high degree of molecular orientation, high mechanical strength, high crystallinity, and high surface area decorated with hydroxyl groups, CNCs were widely applicable in different purposes such as catalysis^11^, papermaking applications^12^, reinforcing filler for polymers^13^, bio-sensing^14^ and bio-imaging^15,16^. Addition of CNCs was approved to not only enhance the hardness of the polymer composites but also enhance the shape memory characters of the polymer matrix^17^, whereas, shape memory polymers (SMPs) are identified as a resilient compounds that could recover their original shape from a temporarily deformed shape after exposing to heat or light or water or changing pH..etc.… CNCs clustered from natural resources were reported to exhibit the capability for forming a percolating network within the polymer matrix to be applicable as reinforcing filler for self-healing polymers^18,19^. CNCs could act as a stabilizer in food industry as solid particles that could be accumulated at oil–water interface to give “Pickering emulsions”^20–22^.

CNCs showed high potentiality for food packaging owing to they can enhance the thermal, mechanical, and barrier characters of the other bio-based compounds with otherwise poor characters. Particularly, the barrier character exhibited with the food packaging compounds acts in preventing food from direct contacting with moisture, microbes or oxygen^23^. The addition of CNCs could improve the water vapor and the oxygen barrier capabilities of the biological food packaging compounds. Some of recent researching approaches were considered with the utilization of CNCs as a drug carrier in the pharmaceutical industry via the hydrophobic association with drug, the covalent attachment of drug, direct binding with drug, encapsulating drug, etc. In recent years, new reports were considered with controlling the drug release at the molecular level via the attachment between the ionized drug moieties and negatively charged CNCs^24,25^. It was demonstrated that CNCs were capable of binding significant quantities of the ionized hydrophilic antibiotics such as tetracycline and doxorubicin that were completely released in 24 h^26^.

Fluorescent gels superiorly exhibit an additional advantage of being light emitting, that endows florescent polymer gel with superior applicability in bio-imaging, photonics, the chemical and environmental sensing. Fluorescent gels are identified as soft gels that are polymeric in nature or composed of small molecular gels through supramolecular assembly of a well-oriented complexes, that are dynamically and reversibly attached through either covalent or noncovalent interactions^27^. Such type of gels could be differed from the conventional hydrogels (swollen with water) or organic gels (swollen with organic solvent) in their light emitting properties with retention of their extended networks. Particularly hydrogels can be applicable as three-dimensional scaffolds for tissue engineering^28^ owing to their flexibility and water retention capability, similar to the biological tissue^29–36^. Additionally, most of gels are not dissolving in water^37^. However, the hydrophilicity renders types of gels able to hold large quantities of water in their highly stable networks. On the other side, hydrogels are widely applicable as biologically active materials in many purposes, such as drug delivery, tissue scaffold.. etc^38–41^. Whereas, they should exhibit antimicrobial performance in order to diminish the risk of microbial infections, which can cause dangerous health problems. Antimicrobial hydrogels can be successfully applicable in the treatment of wounds, to prevent the infection, and heal burnings, cuttings, and scrapes^42,43^. Antimicrobial hydrogel can prevent or slow down the development of microbial infections, which is one of the serious problems that is related to the wound healing as one of the most prominent effects that prevent wound from healing, particularly chronic types, requiring challenging treatments.

Carbon quantum dots (CQDs) are ascribed as a type of carbon nanostructures that were characterized with high-water solubility, low toxicity, preferable biocompatibility, superior photo-stabilization, high sensitivity and selectivity^44–46^. Non-toxicity and biocompatibility of CQDs are identified as prerequisite properties for the application in various biomedical fields, whereas, CQDs nucleated from natural carbon resources were shown to exhibit excellent fluorescent activities and biological properties such as antioxidizing, antitumor and anti-inflammatory impacts^47–51^.

Recent approaches were considerably studied the affinity of various types of biopolymers for fabrication of hydrogels^52–57^. However, in accordance to our knowledge, there are no research approaches that have considered the preparation of microbicide/florescent hydrogel via successive impregnation of CQDs within chitosan as a biocompatible polymer. In the current approach, CQDs and nitrogen containing CQDs (NCQDs) were clustered from CNCs and cationic CNCs (CCNCs), respectively. Preparation of cationic CNCs was performed via grafting with poly-di-allyl dimethyl-ammonium chloride (CNCs-g-poly-DADMAC) via free chain polymerization reaction. Eventually, both of CQDs & NCQDs were impregnated within chitosan as biocompatible polymer for preparation of the required microbicide/florescent hydrogels (CQDs@Chs hydrogel & NCQDs@Chs hydrogel). Successive nucleation of both CQDs & NCQDs was confirmed via several instrumental analyses, like, UV–Visible spectroscopy, Transmission Electron Microscopy, FTIR, ^1^HNMR, ^13^CNMR and photoluminescence. Sequentially, the topographical features, elemental analysis and antimicrobial affinity of the prepared hydrogels after impregnation of the formerly clustered quantum dots within chitosan were currently investigated.

## Experimental work

### Materials and chemicals

CNCs were prepared from wood sawdust according to our previous work^58,59^. The monomer di-allyl dimethyl-ammonium chloride (DADMAC, 65 wt%), sodium hydroxide, sulfuric acid and potassium persulfate (KPS) were purchased from Sigma Aldrich and all chemicals were used as received.

### Preparation of cationic CNCs (CCNCs)

In 150 mL distilled water, 1:1 molar ratio of CNCs and DADMAC were mixed and stirred vigorously at constant 1000 rpm using magnetic stirrer. Temperature was then raised up to 70 °C after the initiator KPS (3 wt% of monomer) was added gently for 1 h. After completing of reaction, 70% ethanolic water was added with continuous stirring until precipitation occurs. The supernatant was collected using centrifuge (10,000 rpm for 30 min) and drained several times with ethanol and finally dried using freeze-drier prior to characterization.

### Synthesis of CQDs & NCQDs

Under magnetic stirring for 30 min at 100 °C, 10 g/L CNCs were separately hydrolyzed with 100 g/L of NaOH. Subsequently, under hydrothermal conditions, in vertical hydrothermal autoclave reactor, the colloidal solution of alkali hydrolyzed polymer was transferred in oven for six hours to complete the successive clustering of CQDs. In order to prepare NCQDs, CCNCs was exploited instead of CNCs. The hydrothermal reaction was performed at low temperature (180 °C) for 5 h, as the reaction temperature shouldn’t be extremely high (> 300 °C) to ensure the complete carbonization of molecular precursor to avoid the deterioration of the polymeric matrix for CNCs as it could result in bad surface passivation with denaturation of their functional groups. Eventually, the prepared colloidal solution with its dark brown color was left for cooling in the atmospheric air to be dialyzed with distilled water using pur-A-lyzer dialysis kit (MWCO 6–8 kDa from Sigma-Aldrich) for ultra-filtration to produce mono-dispersed/purified CQDs for further analysis.

### Preparation of CQDs@Chs & NCQDs@Chs hydrogels

Chitosan (0.4 mg/mL) was gently dissolved in 100 mL of the as-prepared CQDs and NCQDs, separately and pH was adjusted to 6 by using 0.1% acetic acid solution. The former solutions were allowed to stir overnight at ambient temperature. After complete dissolution, the solutions were passed through a syringe filter (0.45 µm) for removing of any impurities. To obtain the hydrogel, the later solutions were then poured in 24-well plates followed by frozen in refrigerator for lyophilizing using freeze-drier at 0.1 mL bar under -60 °C. Physical gelation of chitosan in acetic solution allows the inter- and intra-hydrogen bonds to interact for crosslinking between chitosan molecules and cellulose. By applying the freeze-drying technique, the solution transforms to solid sponge-like structure.

### Characterization and Instrumental analyses

Identification of the chemical structures of CNCs, CCNCs, CQDs &NCQDs were investigated via different spectral mapping results. Spectra were obtained from Jasco FT/IR 6100 spectrometer using attenuated total reflection (ATR) unit. Absorption spectra were analyzed at 500–4000 cm^–1^ using 15 points smoothing, 4 cm^–1^ resolution, 64 scanning times with scanning rate of 2 mm sec^-1^. Nuclear magnetic resonance (^1^H-NMR and ^13^C-NMR) were estimated from Jeol‐Ex‐300 NMR spectrometer (JEOL – Japan). For identification of the effect of the performed synthesis method on the degree of crystallinity of CNCs and CCNCs, the analysis of powder X-ray diffraction was carried out using X’Pert MPD diffractometer system from Philips, at room temperature. The diffraction bands were detected in the diffraction angle (2θ) range of 3.5–50° using monochromator (Cu Kα X-radiation at 40 kV, 50 mA and λ = 1.5406 Å). Geometrical features and size average of the ingrained CQDs were estimated via anticipation of High-Resolution Transmission Electron Microscope from Japan (JEOL-JEM-1200). Size average for both of CQDs & NCQDs was estimated by 4 pi analysis software (from USA) for at least 50 particles. The optical activity was followed up via the investigation of photoluminescence for CQDs & NCQDs in ultraviolet–visible range was estimated via Spectro-fluorometer (JASCO FP8300). Spectral data were analysed at room temperature with excitation at 340 nm. The quantum yield of the synthesized CQDs & NCQDs were measured according to literature^60^. While, the quantum yield was estimated relative to fluorescent Rhodamine 6G with known quantum yield of (95%). Rheological properties were investigated by measuring the viscosity using B-ONE Plus Viscometer from Lamy Rheology instruments. The measurements were carried out using rode of L4 at 100 rpm for 15 s.

The comparative method of Williams et al., which involves the use of multiple well characterized references with known fluorescence quantum yields. It could be expressed as a time consuming method, however, it provides high accuracy by calculating the slope of the line generated by plotting the integrated fluorescence intensity against the absorption for multiple concentrations of fluorophore. In this technical note, the comparative method was applied to determine the fluorescence quantum yield of Rhodamine B by comparison to Rhodamine 6G which has fluorescence quantum yield of 0.95. Results from both the comparative and the single-point methods are compared and evaluated against the well-known value of the Rhodamine B.

### Microbicide performance

Microbicide performance of the prepared CQDs@Chs & NCQDs@Chs against the selected pathogens was examined via the qualitative methodology of inhibition zone^61^. In this procedure, three different microbial species of gram-positive bacteria (*Staphylococcus aureus*), gram-negative bacteria (*Escherichia coli*) and fungal species (*Candida albicans*) were tested. In the inhibition zone method (disk diffusion test), the different tested bacterial species were grown in the medium for the preparation of the microbial suspension. 100 μL of microbial colloid was diffused on the agar plate for broth in which it was maintained. CQDs@Chs & NCQDs@Chs (0.5 cm) were added in the middle of the plate to be incubated at 37 °C for 24 h. The diameter of the inhibition zone was evaluated in millimeters using slipping calipers according to NCCLS, 1997^61^. After incubation, the colony forming units (CFU) were counted for each plate^61,62^.

## Results and discussion

### Preparation of CNCs-g-Poly-DADMAC (CCNCs)

**Scheme 1:**
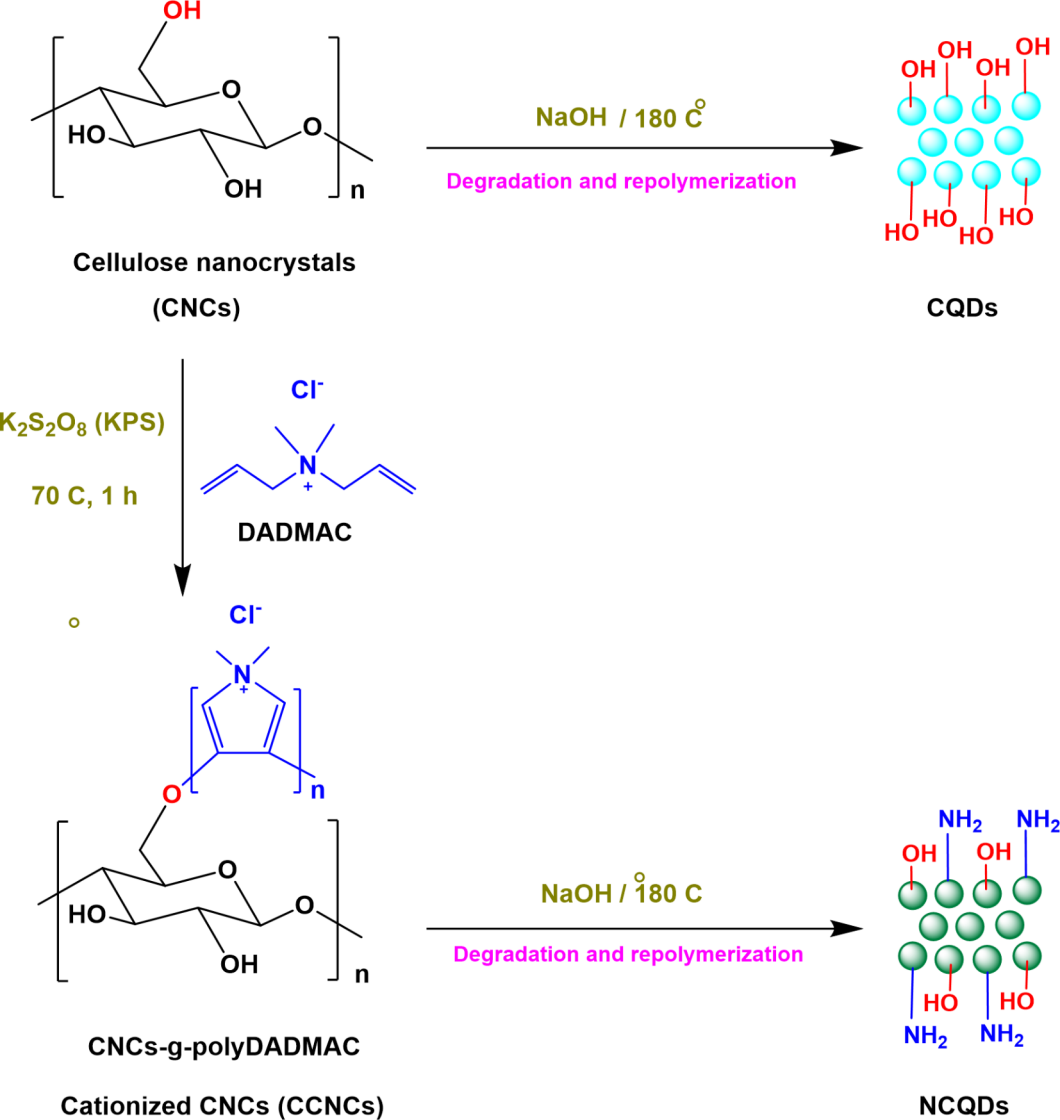
The schematic preparation of CCNCs, CQDs and NCQDs.

The current approach uniquely investigated the successive nucleation of NCQDs from CCNCs. The diagram in Scheme 1 represents a tentative mechanism for preparation of CCNCs with quaternary amine for preparation of CNC-g-poly-DADMAC (CCNCs), as it was achieved through a novel approach that the surface modification of CNCs was proceeded via free radical polymerization of DADMAC monomer in the presence of KPS as initiator^63,64^. The graft polymerization reaction was conducted for 1 h at 70 °C and the graft ratio (GR) % was determined according to Eq. (1), where, Wb (g) is the grafted polymer (CCNCs) weight and Wa (g) is CNCs dried weight. In this context, GR% of the resultant CCNCs was reached 12.4 ± 0.2% which, in turns, emphasize the chemical modification of CNCs surface by 12.4% of poly-[DADMAC] (76.9 mol/g).

1 $${\text{GR}}\% = \left( {{\text{Wb}}{-}{\text{Wa}}} \right)/{\text{Wb}} \times {1}00$$

GR; grafting ratio (%), Wa and Wb refer to the weight before and after grafting (g).

#### Characterization of CNCs & CCNCs

**Fig. 1:**
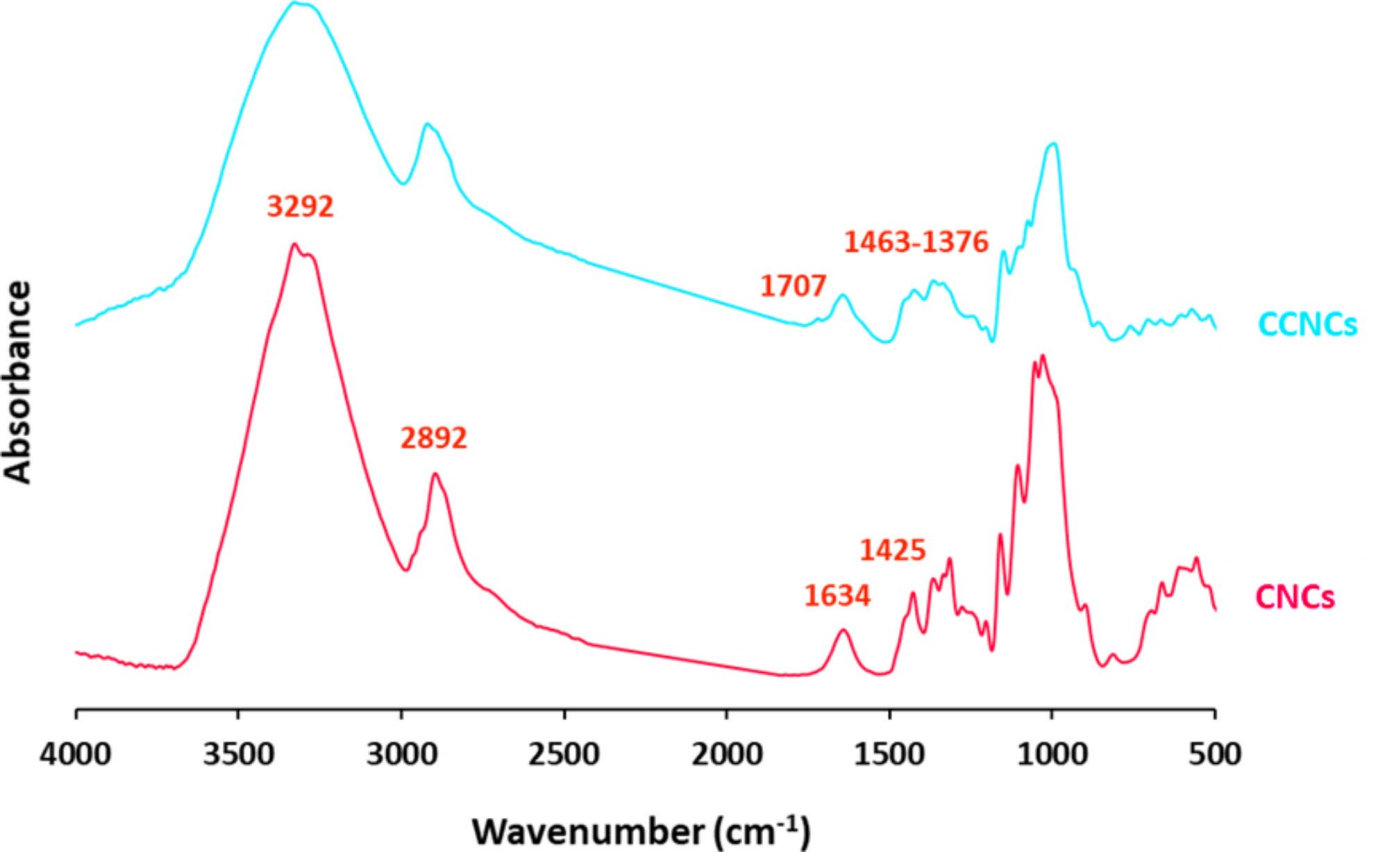
FTIR spectra for CNCs and CCNCs.

**Fig. 2:**
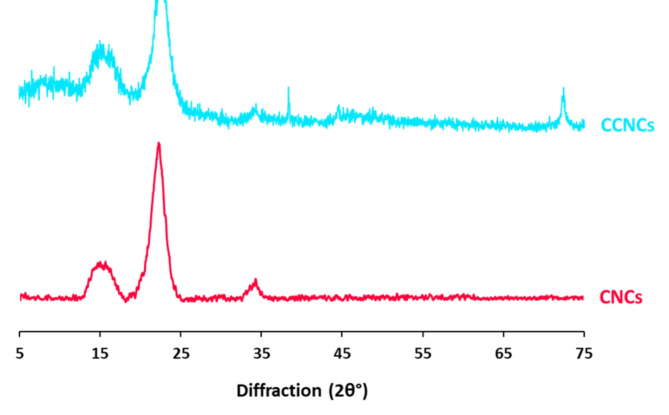
XRD patterns for CNCs and CCNCs.

Figure 1 represents FTIR spectral data for CNCs & CCNCs. In order to identify the interchanging in the chemical composition of cationic CNCs, from the plotted data it could be declared that, CNCs were characterized with significant bands at 3292 cm^−1^, 2892 cm^−1^, 1634 cm^−1^, 1425 cm^−1^, 1366–1272 cm^−1^, 1041–1122 cm^−1^ and 1021 cm^−1^ that are characteristic for O–H stretching, symmetric vibration of aliphatic C–H, C = O stretching, bending vibration of C–H, O–H bending, characteristic band glycoside linkage and vibration of C–O, respectively^65,66^. Moreover, spectrum of CCNCs showed that, for cationic CNCs, the as-mentioned characteristic bands were retained but with lower intensity and additional new bands at 1707 cm^−1^ assigned for N–H bending. On the other hand, NMR spectral data were plotted in Figure S1 (Supplementary file) for CNCs and CCNCs. From Figure S1a &S1b that presented ^1^HNMR for CNCs & CCNCs, respectively, it could be obviously observed that, the characteristic peak for protons attached to C–O (5 ppm) was retained after cationization for CCNCs in addition to that corresponding to protons of N–H amine (1–2 ppm). Figure S1c &S1d showed ^13^CNMR spectra for CNCs & CCNCs, as all of the typical bands for CNCs (62 ppm for sp^3^ carbons & 180 ppm for carbons of C = O groups) were detected. The as-produced CCNCs were exhibited with significant bands of sp^3^ carbons at 62 ppm. Carbons attached with hydroxyl groups at 73 ppm, C = C for aromatic or sp^2^ carbons at 172 ppm and for carbons of C = O groups at 180 ppm, were all observed in the represented spectral map^67,68^. Moreover, Fig. 2 for XRD analysis shows that, for CNCs, significant peaks for cellulose I, cellulose II corresponding to the (020) plane and (200) plane were indexed at 2θ around 15.7°, 22.6° and 34.7°. Whereas, for CCNCs, the as-mentioned bands were retained in addition to new peaks were estimated at 2θ around 38.4° & 72.5° corresponding to (111) & (311) planes.

#### Characterization of CQDs & NCQDs

Successive clustering of either CQDs or NCQDs was performed under basic conditions via hydrothermal technique. Herein, the affinity of both CNCs & CCNCs for controllable clustering of CQDs & NCQDs as extrinsic fluorophores to be sequentially exploited in preparation of microbicide/florescent hydrogels is approved in the current approach. Accordingly, the clustering mechanism of CQDs from CNCs could be predicted as follows; clustering was performed via two main steps; carbonization and surface passivation (surface functionalization). In carbonization, either CNCs or CCNCs act as the molecular precursors to produce basic carbon containing residues that are subsequently nucleated under hydrothermal conditions giving the required QDs. While, the passivation as the eventual step minimizes the effects of the surface-defect, trap sites, and the direct quenching from the surrounding media, for improving of photoemission. Whereas, the pure nucleated carbon nanoparticles are well known to be optically inactive. Moreover, surface decoration of CQDs (hydroxyl groups) or NCQDs (nitrogen and oxygen containing groups), acted in insulating the surface of carbon nanostructures. In accordance to literature^69–71^, excellent optical properties of QDs could be acquired via surface passivation with heteroatom containing decorative groups. Dialysis was lastly performed for ultra-purification to ingrain highly purified/mono-dispersed CQDs with controllable geometrical features^72^. The quantum yield of the synthesized CQDs and NCQDs was estimated to be 28% and 33%, respectively.

**Fig. 3:**
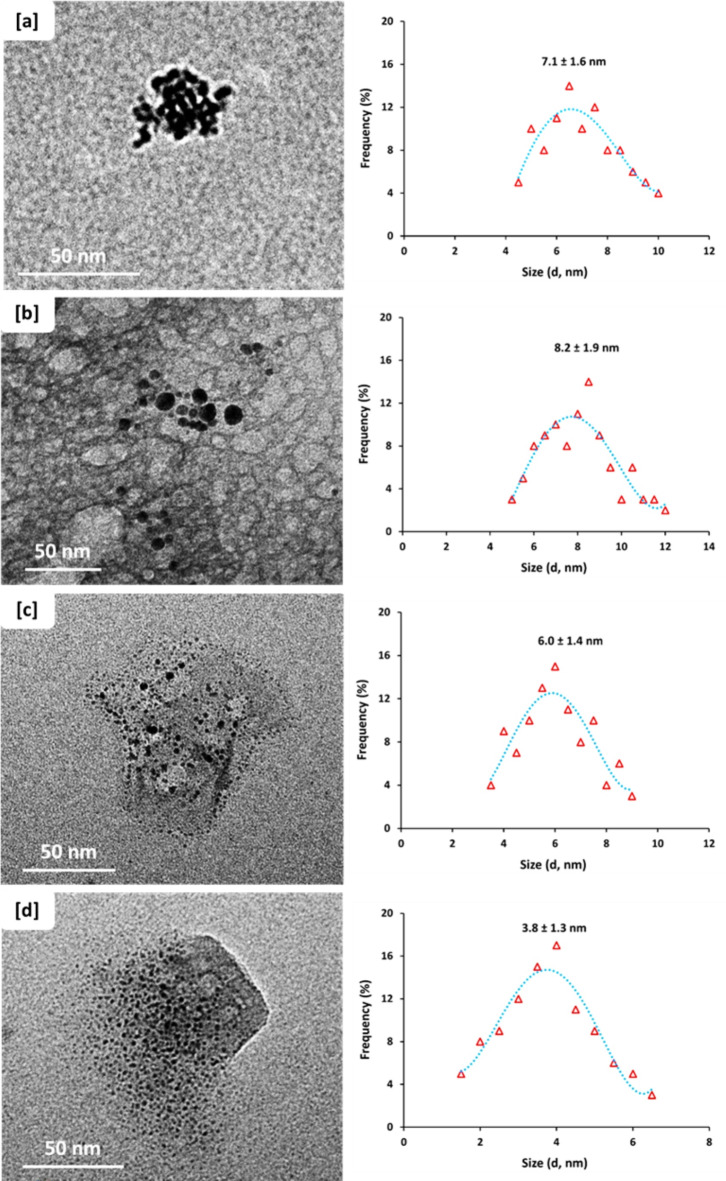
TEM micrograph and size distribution for the prepared CQDs; **[a]** CNCs-0.5%, **[b]** CNCs-2.0%, **[c]** CCNCs-0.5% and **[d]** CCNCs-2.0%.

To show the topographical features and to estimate the size distributions; Fig. 3a, b represented TEM micrographs for CQDs ingrained from CNCs with two different concentrations of 0.5% & 2%, respectively, whereas, Fig. 3c, d represent TEM images for NCQDs ingrained from CCNCs with two different concentrations of 0.5% & 2%, respectively. From the plotted micrographs, the compatibility was affirmed for both CNCs & CCNCs and their vital role in successive nucleation of spherical/highly mono-dispersed QDs. From the estimated data, it could be depicted that; increment the concentration of either CNCs or CCNCs from 0.5% to 2% resulted in slight enlarging of the particle size for the dispersed QDs.

The size distribution of CQDs ingrained from CNCs with concentration of 0.5% & 2% was increased form 7.1 ± 1.6 nm (Fig. 3a) to 8.2 ± 1.9 nm (Fig. 3b), respectively. However, the particle size and topographical features of QDs were exhibited to be more controllable when it was successively ingrained from CCNCs to be estimated with size average of 6.0 ± 1.4 nm (Fig. 3c) when the concentration of the colloidal solution of CCNCs was 0.5%. Similarly, increment of CCNCs concentration up to 2% resulted in nucleation of NCQDs with size distribution of 3.8 ± 1.3 nm (Fig. 3d). These could be attributed to the fact that, CCNCs with concentration up to 2% under the effect of hydrothermal conditions could act in improving the polymer accessibility to generate higher amounts of alkali fragmented moieties that could superiorly exploited for nucleation of smaller sized NCQDs, as reported in literature^68,73,74^.

**Fig. 4:**
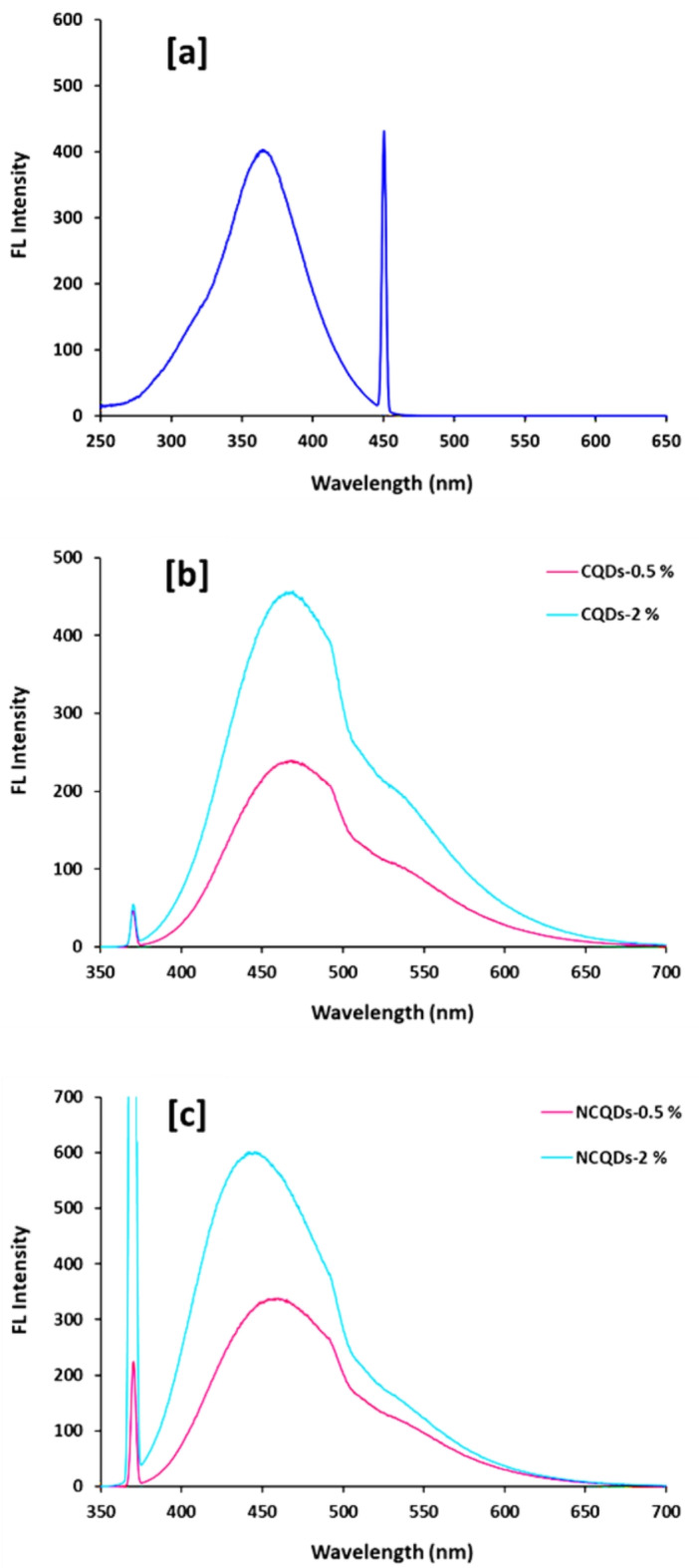
Excitation-emission spectra for the prepared CQDs at room temperature; **[a]** excitation at 450 nm for CNCs-2.0% and **[b, c]** Emission spectra (excitation at 370 nm) for the prepared CQDs samples.

Figure 4a represents the excitation spectra for CCNCs (2%) at 450 nm and showed that there was a broad excitation peak at 370 nm. Therefore, the emission of samples was detected at the excitation wavelength of 370 nm. Figure 4b, c are plotted for the emission spectra of CNCs & CCNCs with two concentrations 2% & 0.5%. From the presented spectra, it could be declared that, CCNCs were showed to significantly enhanced optical activity, whereas, CCNCs were exhibited with higher intense emission bands compared to that of CNCs. Moreover, higher concentration of CNCs & CCNCs (2%) exhibited with higher florescent intensities, as it was estimated to be 454 & 598 (a. u.) at particular wavelengths of 470 nm corresponding to the green region^67,74–77^, for CNCs & CCNCs, respectively. Exhibition of strong fluorescent bands in visible range will esuriently facilitate their applications in biomedical purposes and has currently elaborated by the photoluminescence spectra of both CNCs & CCNCs.

**Fig. 5:**
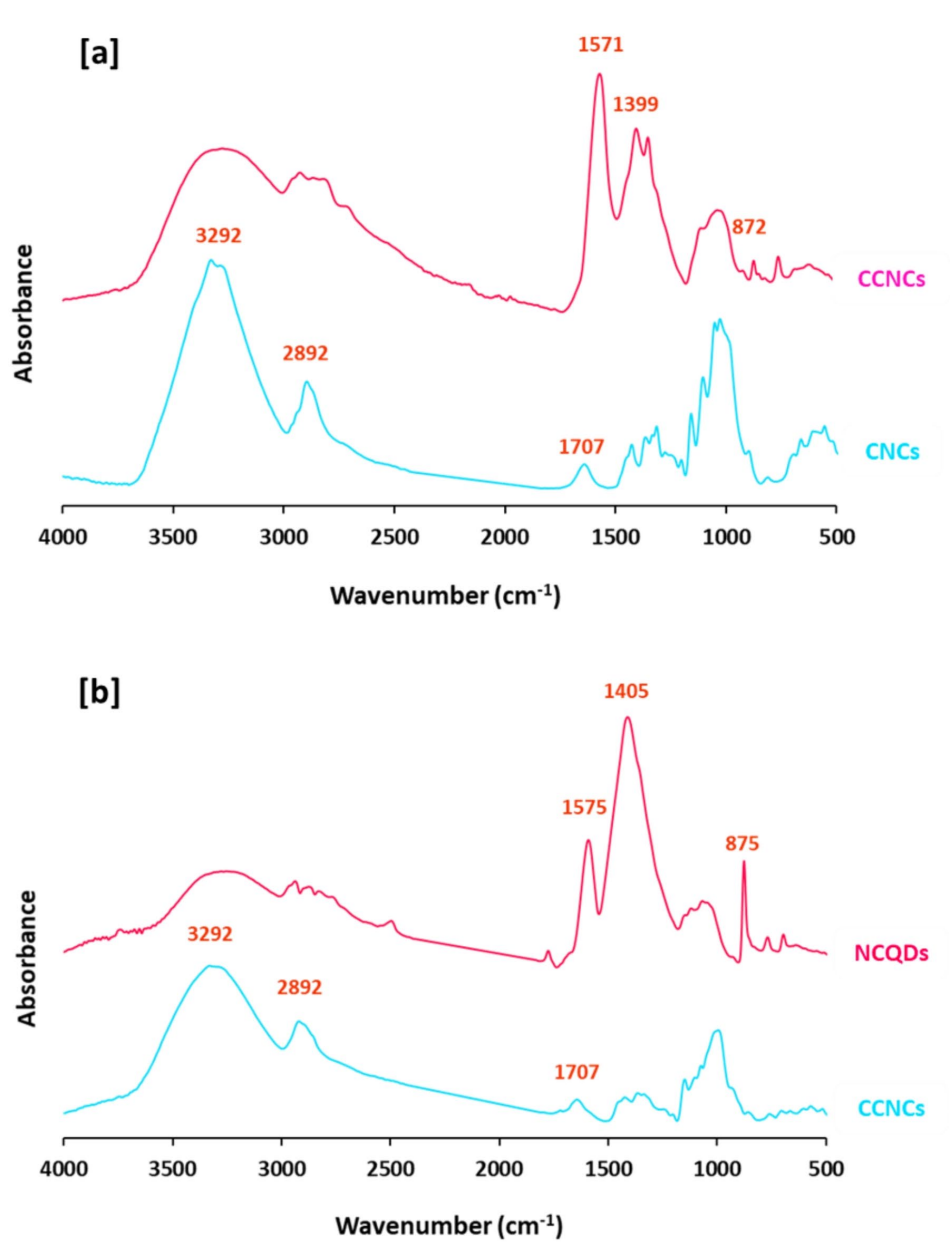
FTIR spectra for the prepared CQDs.

Figure 5 represents FT-IR spectral mapping results of CQDs and NCQDs. After functionalization of CNCs & CCNCs for successive clustering of CQDs & NCQDs, the characteristic band for aliphatic C-H was shown with significant lower intensity and the band of C = O was shown with observable high intensity and shifted to lower wavenumber (1571/1575 cm^-1^). Moreover, two bands were estimated at 1405/1399 and 875/872 cm^-1^ which are corresponding to C–C aromatic ring and aromatic sp^2^ C–H bending, respectively^68,74^.

**Fig. 6:**
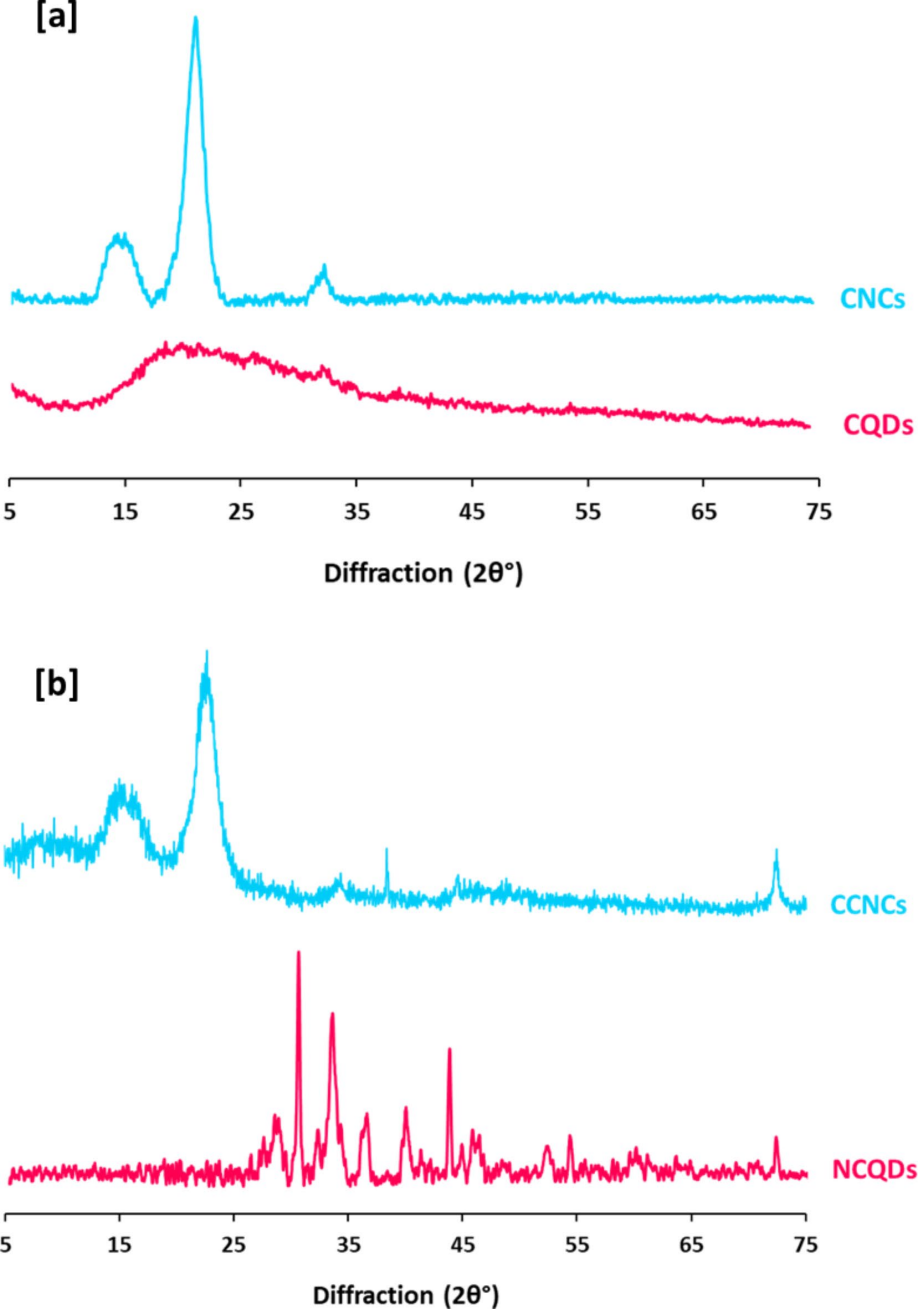
XRD patterns for the synthesized CQDs and NCQDs.

On the other hand, NMR spectral data were plotted in Figure S2 for CQDs and NCQDs. It could be obviously observed that, showed the characteristic bands for protons of sp^3^ C-H at 1–2 ppm, protons of O–H at 3.5–4 ppm, protons bonded to C = O groups at 4.5–6 ppm and aromatic or sp^2^ CH = CH protons at 8–8.5 ppm, were all similarly detected for spectrum of NCQDs. Figure S2c & S2d showed ^13^CNMR for CQDs & NCQDs, respectively, whereas, all the typical bands for CNCs (at 0–60 ppm corresponding to sp^3^ carbons, 73 ppm signified for carbons attached with hydroxyl groups, 165 ppm C = C specialized for aromatic or sp^2^ carbons, 173 ppm corresponding to carbons of carboxylic acid groups and 182 ppm characteristic for carbons of C = O groups) were similarly estimated for NCQDs with more intense^67,68^. Figure 6 shows the analyzed data of XRD that were detected for CQDs & NCQDs. Figure 9a declares that, significant peaks for CNCs were disappeared after its exploitation in clustering of CQDs, while, the significant broadly band for cellulose I was detected. After nucleation of NCQDs, the previously mentioned bands of CCNCs were disappeared and new peaks were estimated at 2θ = 29.8°, 31.7°, 34.7°, 38.1°, 41.6°, 45.5°, 48.1°, 54.6°, 55.6° & 75.4° indexed for (210), (122), (200), (231), (142), (241) and (220) planes^78,79^.

#### Fluorescent CQDs@Chs & NCQDs@Chs hydrogels

Fluorescent activity is the process of emitting light via a certain material (like polymer with intrinsic fluorescence, quantum dot, fluorophore dye) that could absorb the light at a higher energy (lower wavelength) and emits light of lower energy (longer wavelength). Once the source of light is removed, fluorescent activity could stop within nanoseconds. The fluorescence quantum yield is the ratio of the number of the emitted photons to the number of the absorbed photons, while, the fluorescence lifetime is the average time that a fluorophore takes in the excited state before the emission of photons. The mechanism of emission for the different fluorescent moieties impregnated within gels could be illustrated as follows; the mechanism could be illustrated via the Jablonski diagram, as via Förster (or fluorescence) resonance energy transfer (FRET), the energy of fluorescence could be transferred between two chromophores that are close to each other in physical proximity^80^.

In this process, from an excited donor chromophore to an acceptor chromophore, the energy is transferring through non-radiative dipole–dipole coupling. Fluorescent moieties can be generally categorized as extrinsic or intrinsic fluorophores. Intrinsic fluorophores are ascribed as naturally existing compounds, like the green fluorescent proteins. Whereas, Extrinsic fluorophores are often being synthetic nanostructures, like quantum dots (QDs). The later are investigated as semi-conducting nanostructures and the color and affinity of their fluorescent activity is strongly size-dependent with potentially high quantum yields and sharp emission bands^81,82^. The nano-dimensions of quantum dots facilitate their impregnation into the supramolecular network, such as polymeric hydrogel. Carbon counterparts that are exploited in the gels, ascribed as carbon quantum dots, are carbon-based fluorescent nanostructures, could emit photons as a result of the quantum confinement effects as a result of their nano-size^83–89^.

The optical activity of CQDs was mainly correlated to the surface passivation to be decorated with accessible functional groups^90^. Wu et al. confirmed that, the optical activity of QDs is principally dependent on the surface state and its decoration with functional groups on graphite sheets^34^. The surface state is ascribed as the optically active center that is composed through the synergetic hybridization of the carbonic cores with functional groups^91^. Characteristic biomaterials as soft gels are ascribed as formidable reagents for biomedicine and tissue engineering. Fluorescent gels based on biopolymers are additionally characterized with emitting light, endowing this type of materials with advanced applicability in chemical and environmental sensing, bio-imaging and photonics^92^.

The current study represents a unique fabrication strategy for preparation of fluorescent gels via incorporation of the as-prepared florescent QDs (CQDs & NCQDs) as fluorophore entities into chitosan as a gelling matrix. According to literature^93–97^ the gel matrix is prepared via self-assembly of chitosan as supramolecular gels, while the currently prepared QDs as the photoluminescent tag molecules are immobilized via either diffusion into the chitosan network or by the orthogonal reaction with the highly branched chitosan matrix for co-assembly. Moreover, it could be assumed that, the impregnated QDs cloud be bonded with the accessible groups of chitosan macromolecules via hydrogen bonding for preparation of highly stabilized hydrogel network.

**Fig. 7:**
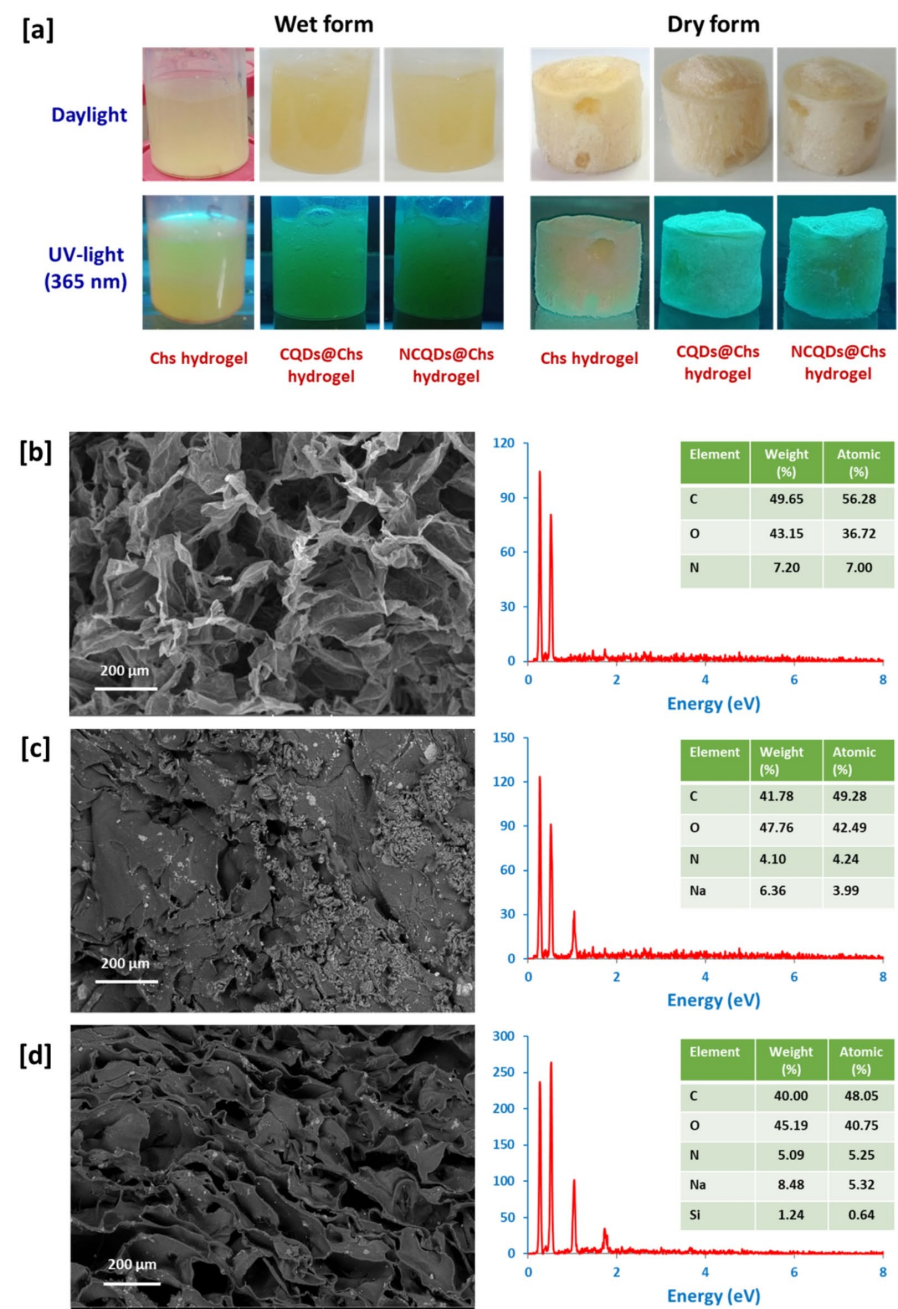
**[a]** photographic images for the hydrogels**. [b-d]** Microscopic images for the synthesized hydrogel; **[b]** Chs hydrogel, **[c]** CQDs@Chs hydrogel and **[d]** NCQDs@Chs hydrogel.

Rheological properties were performed, whereas, the viscosity was estimated to be 1108 mpa, 1386 mpa and 1244 mpa, for Chs, CQDs@Chs and NCQDs@Chs solutions, respectively. The viscosity was gradually increased for Chs solution after addition of CQDs and NCODs solutions; however, the produced hydrogels were quite good and acceptable as seen in Fig. 10a. The showed images of the obtained hydrogels, obviously declared the interchanging of color from creamy white for CQDs@Chs hydrogel & NCQDs@Chs hydrogel at daylight to greenish yellow under UV-lamp (365 nm). The morphological characters of the obtained hydrogels were examined for the cross-section under the electron microscope as clarified in Fig. 7. The Chs hydrogel showed a homogeneous networked structure with well-distributed/irregular pores. The size of the pores was estimated to be 20–50 μm (Fig. 7b). The recorded signals in EDX analysis spectra are referred to the elements of O, C and N which are corresponding to the chitosan molecules. After the impregnation, the particles of CQDs and NCQDs were individually distributed within the micro-pores of Chs hydrogel (Fig. 7c, d). The elemental analysis of QDs is exhibited with the same signals of Chs, with Na signal that is related to the alkaline medium of CQDs and NCQDs. The microscopic observation confirmed impregnation of CQDs & NCQDs particles within the chitosan matrix.

**Fig. 8:**
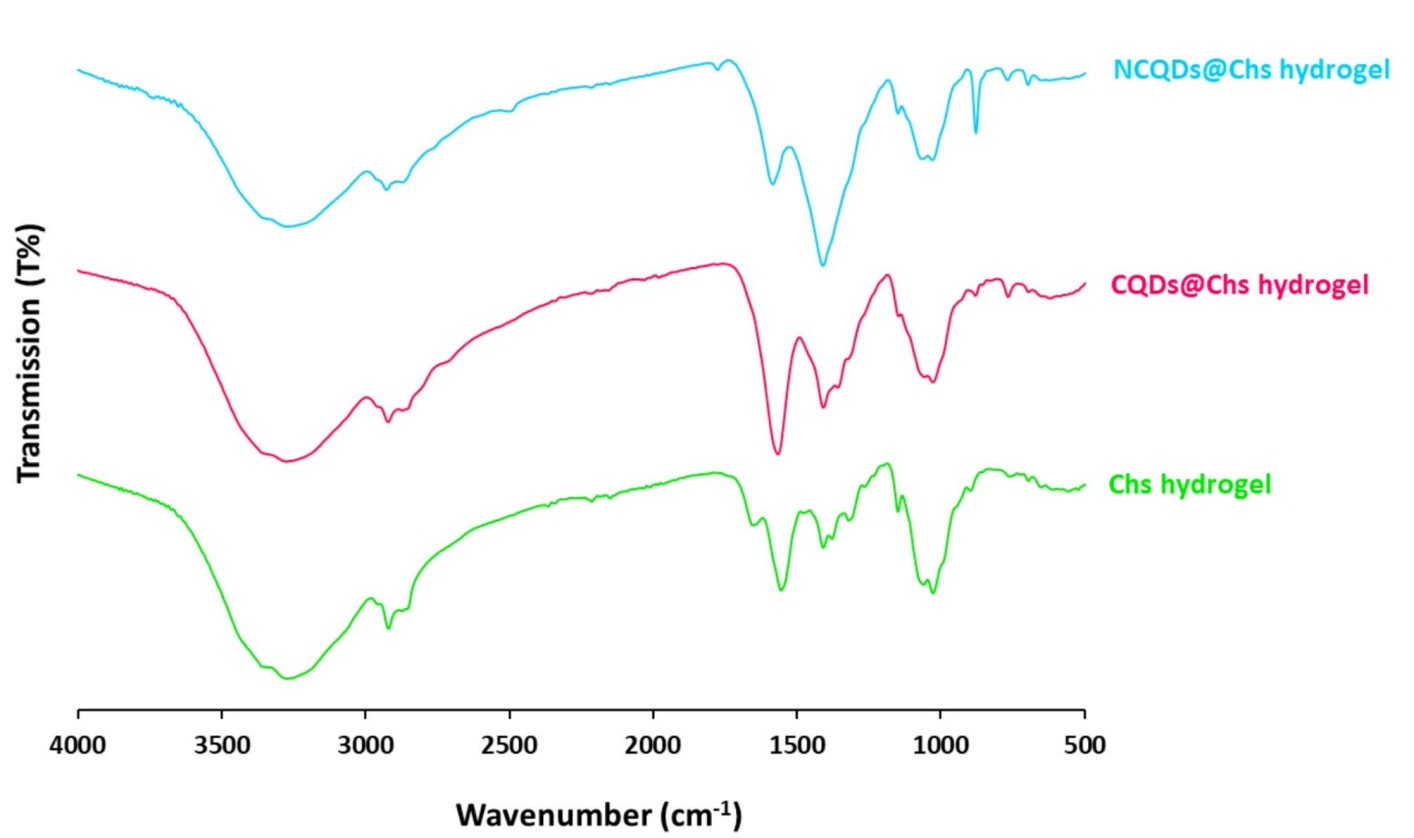
FTIR spectra for the synthesized hydrogels.

The FT-IR spectral results for the hydrogels were presented in Fig. 8. Chs hydrogel exhibited six main spectral bands at 3292, 2918/2851, 1640, 1552, 1403–1364, 1025 cm^-1^. The recorded bands are assigned for N–H/O–H stretching, aliphatic C-H vibration, C = O stretching, C-N stretching, N–H bending and glycoside linkage of C-O, respectively. These bands are characterized for the chitosan macromolecules^98,99^. After impregnation of CQDs and NCQDs, three new bands are appeared at 1571, 1405/1399 & 875 cm^-1^ and 1589, 1410, 877 cm^−1^, respectively. The appeared new bands are corresponding to CQDs and NCQDs which are successfully inserted within the Chitosan hydrogel network.

#### Microbicide potency of hydrogels

**Fig. 9:**
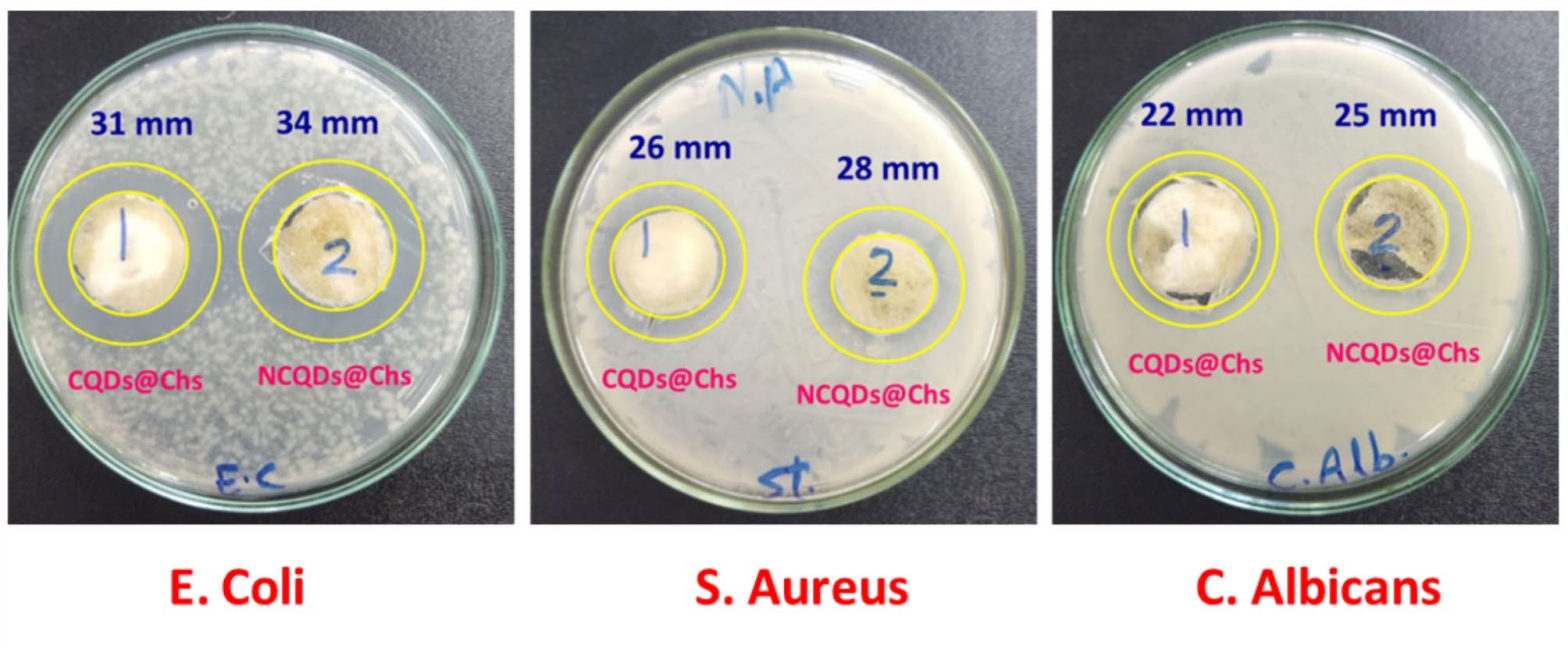
Antimicrobial activity for the synthesized hydrogel via inhibition zone evaluation.

The porous structure of hydrogel can be ascribed as an excellent matrix for loading of antimicrobial laborers such as antibiotics, extracts, nanoparticles, etc.… Also its structure is suitable for controllable releasing rate of the antimicrobial agent^54,100^. According to literature, CQDs were shown with superior microbicide potentiality against different microbial species to show its compatibility for preparing hydrogels doped with CQDs to be used as antimicrobial biomaterials, particularly wound dressings^101–108^. In the current study, using inhibition zone technique, the microbicide potentiality for CQDs@Chs & NCQDs@Chs hydrogels was studied against three different microbial species; gram positive bacterial strains (*Staph. aureus)*, gram negative bacterial strains (*E. coli*) and fungal strains (*C. albicans*). The proceeded experimental work strongly revealed that, against all the examined microbial strains, both of CQDs@Chs hydrogel & NCQDs@Chs hydrogel showed excellent microbicide potency (Fig. 9). By exploiting CQDs@Chs hydrogel, the diameter of the inhibition zone in the plates prepared with *Staph. aureus, E. coli* and *C. albicans*, was estimated to be 26, 31 & 22 mm, respectively. Whereas, the application of NCQDs@Chs hydrogel was shown significantly higher antimicrobial performance, whereas, the inhibition zone was estimated to be 28, 34 & 25 mm for plates prepared with microbial strains of with *Staph. aureus, E. coli* & *C. albicans*, respectively.

Meanwhile, the estimated results are in harmony with that studied in literature^101,104,106,107,109^. The mechanism of microbicide action for the prepared CQDs@Chs hydrogel & NCQDs@Chs hydrogel could be simply described as follows:

i) According to literature, chitosan could exhibit antimicrobial action at pH below 6, whereas, the amine groups as terminal functional groups for chitosan building blocks are positively charged and thus can interact electrostatically with negatively charged groups on the bacterial membrane^110^. So as chitosan macromolecules can disrupt the normal functionality of the bacterial membrane through provoking the leakage of intracellular ingredients and could inhibit the transportation of the nutrients into the cells^111–113^. However, in the current study, the process of preparing CQDs@Chs & NCQDs@Chs hydrogels was performed at pH adjusted to 6, meanwhile, the antimicrobial action of CQDs@Chs & NCQDs@Chs is only correlated to the doped QDs.
ii) The excellence of antimicrobial performance for the currently prepared CQDs@Chs & NCQDs@Chs hydrogels might be due to the reactive oxygen species (ROS) that were liberated from the heteroatom containing functional groups of both CQDs & NCQDs.
iii) The generated ROS might act in mortal action for the exposed microbial cells, as ROS could adhere, penetrate the microbial cell wall and motivate the oxidative stress with deterioration of DNA & RNA, resulting in the inhibition and altering the gene expressions. Additionally, ROS might act in the mitochondrial dysfunction, per-oxidization of lipid, inactivation of intracellular protein, gradual decomposition of the cell wall, and eventually lead to apoptotic cell death.
iv) NCQDs@Chs hydrogel showed significantly higher microbicide performance owing to the decoration of the impregnated NCQDs with both oxygen and nitrogen containing functional groups, with smaller size than CQDs immobilized within CQDs@Chs hydrogel that is decorated with oxygen containing functional groups. As according to literature, decoration of CQDs with nitrogen containing functional groups showed higher affinity in biological activities, due to the higher affinity of nitrogen in binding with the bacterial surface functional groups for final bacterial death^101,104,106,107,109^. Moreover, the superiority of NCQDs@Chs hydrogel in the antimicrobial performance is logically correlated to the smaller sized NCQDs, so as, more moieties of NCQDs were impregnated within Chs matrix, and in turn more amount of RS were liberated for antimicrobial performance.

## Conclusion

The current study represents unique fabrication strategy for the preparation of fluorescent hydrogels via incorporation of florescent quantum dots (QDs) as fluorophore entities into chitosan as a gelling matrix. QDs categorized as carbon quantum dots (CQDs) & nitrogen containing carbon quantum dots (NCQDs) were preliminary synthesized from cellulose nanocrystals (CNCs) and cationic cellulose nanocrystals (CCNCs), respectively. CCNCs were prepared via grafting with poly-diallyl dimethylammonium chloride (CNCs-g-poly-DADMAC). CQDs & NCQDs were impregnated within chitosan for the preparation of the required microbicide/florescent hydrogels (CQDs@Chs hydrogel & NCQDs@Chs hydrogel). Successive nucleation of both CQDs & NCQDs was confirmed via several instrumental analyses. The data revealed that, CCNCs could be successfully exploited in the preparation of NCQDs with more controllable size and superior photoluminescence. Moreover, increment of CNCs concentration reflected in nucleation of enlarged QDs, at variance of CCNCs, whereas, rising its concentration resulted in significantly smaller sized QDs. Whereas, size distribution of CQDs ingrained from 2% CNCs was estimated to be 8.2 nm, while, NCQDs ingrained from 2% CCNCs exhibited with size distribution of 3.8 nm. The prepared CQDs@Chs hydrogel & NCQDs@Chs hydrogel showed excellent antimicrobial performance in visible light against different pathogens including bacteria and fungi. So, it could be summarized that; CCNCs showed seniority in nucleation of QDs with significantly higher photoluminescence and microbicide activities.

## Electronic supplementary material

Below is the link to the electronic supplementary material.

Supplementary Material 1

## Data Availability

The datasets used and/or analysed during the current study available from the corresponding author on reasonable request.
