# Supplementary material for: Fluorescent antimicrobial hydrogel based on fluorophore N-doped carbon dots originated from cellulose nanocrystals
Source: Sci Rep. 2024 Nov 25;14:29226. doi: 10.1038/s41598-024-80222-7 (PMC11589154; doi:10.1038/s41598-024-80222-7)
Supplement: Supplementary file 1 — Supplementary Material 1 [file 41598_2024_80222_MOESM1_ESM.docx]

**Supplementary file**

**Figure S1:** NMR Spectral data for CNCs and CCNCs; **[a, b]** ^1^HNMR**, [c, d]** ^13^CNMR, **[a, c]** CNCs and **[b, d]** CCNCs.

**
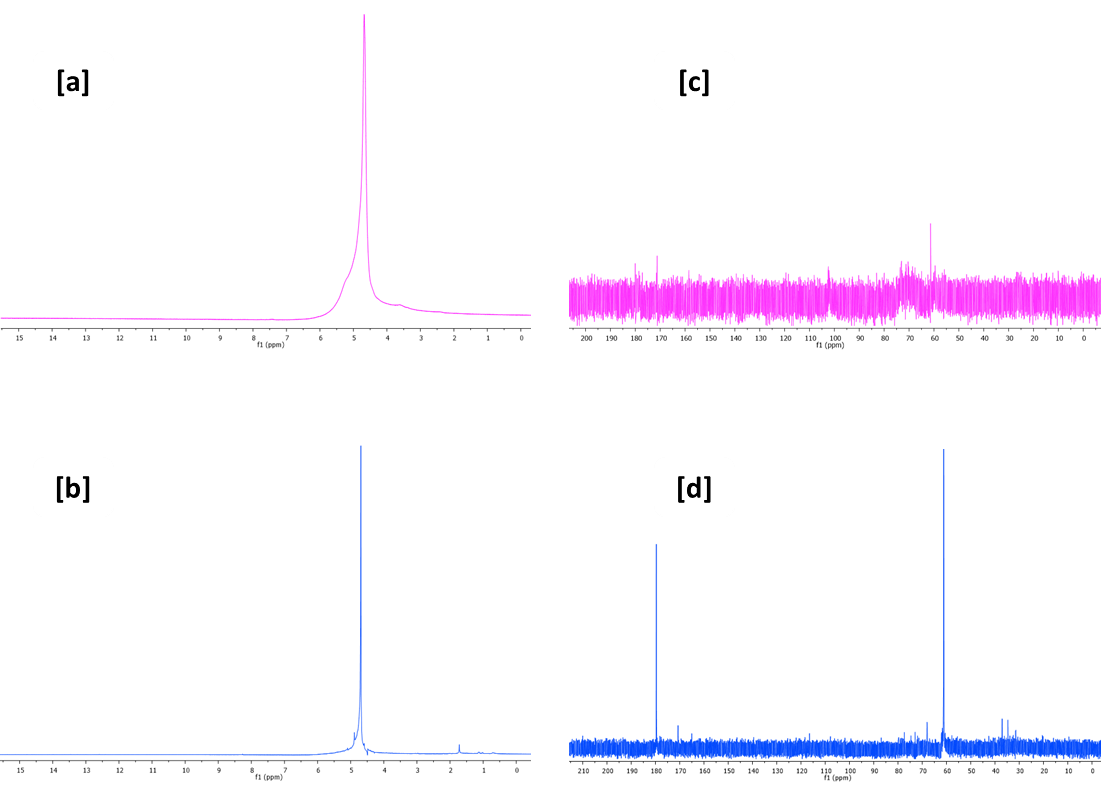
**

**Figure S2:** NMR Spectral data for the prepared CQDs; **[a, b]** ^1^HNMR**, [c, d]** ^13^CNMR, **[a, c]** CQDs and **[b, d]** NCQDs.

**
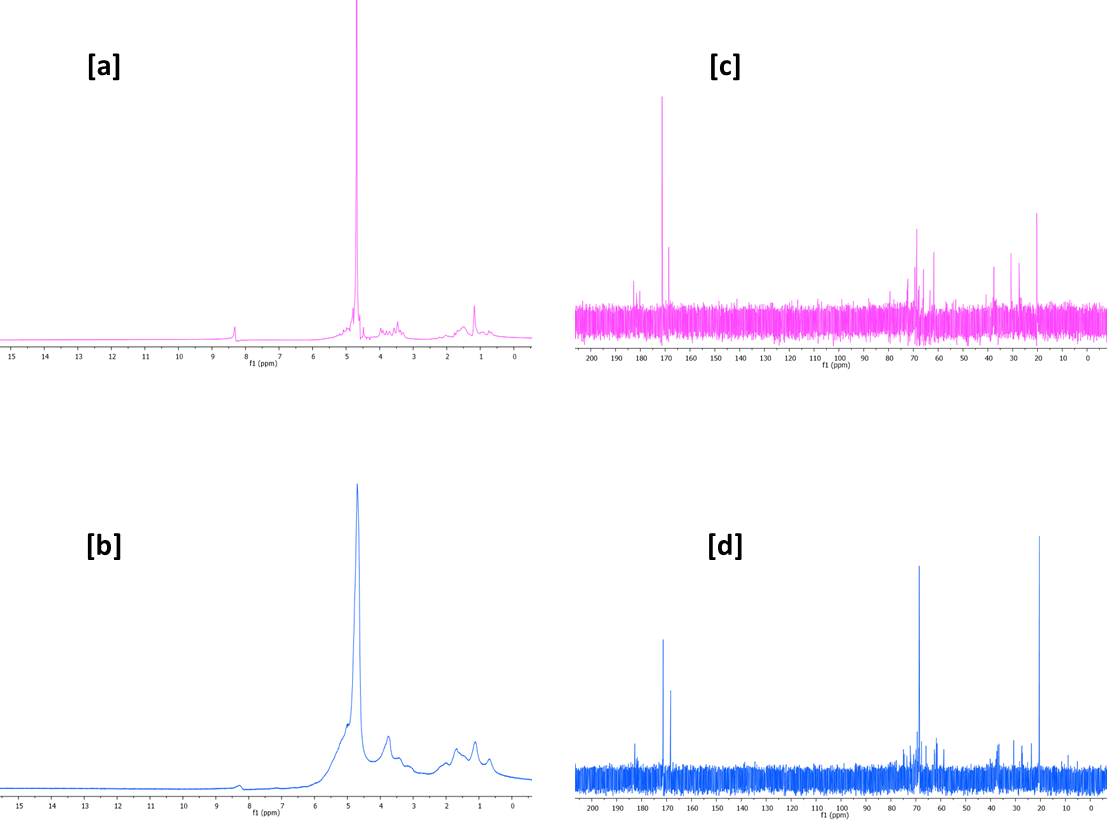
**
